# Communicating health risks in science publications: time for everyone to take responsibility

**DOI:** 10.1186/s12916-018-1194-4

**Published:** 2018-11-13

**Authors:** Alexandra L. J. Freeman, David J. Spiegelhalter

**Affiliations:** Winton Centre for Risk and Evidence Communication, University of Cambridge, Cambridge, UK

**Keywords:** Alcohol, Absolute risk, Relative risk, Press release, Practical significance, Risk communication

## Abstract

Research that is poorly communicated or presented is as potentially damaging as research that is poorly conducted or fraudulent. Recent examples illustrate how the problem often lies with researchers, not press officers or journalists. The quest for publication and ‘impact’ must not outweigh the importance of accurate representation of science; herein, we suggest steps that researchers, journalists and press officers can take to help ensure this.

## Putting medical risk into perspective

Medical researchers are increasingly in search of a newspaper headline; this, coupled with the plethora of traditional and social media outlets hungry for the latest research on a topic ‘relevant’ to their audiences, is proving a match made in heaven. Readership, sales and ‘impact’ all seem to benefit, but do any of us end up at all wiser?

**Fig. 1:**
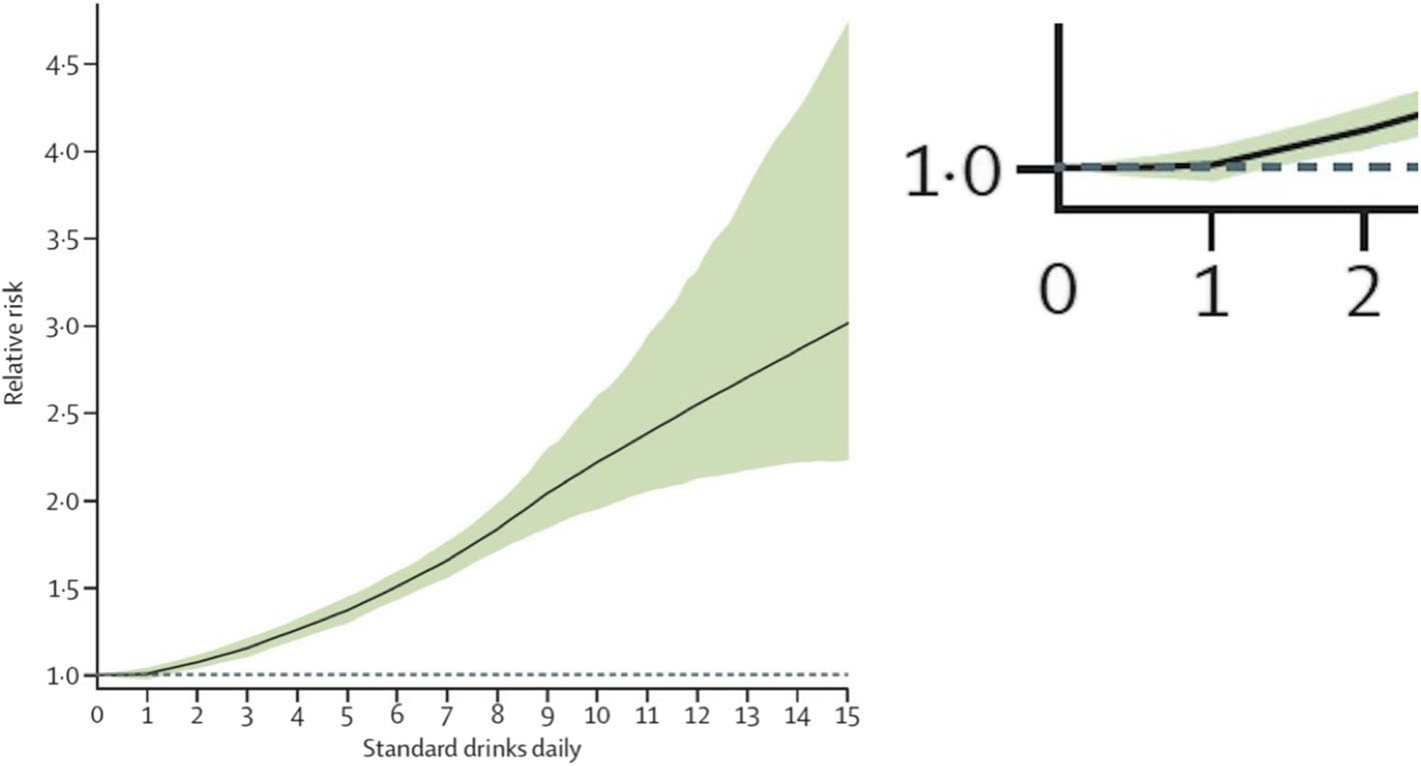
Adapted from Figure five within reference [3]. The expanded section shows the estimated effect at one drink per day, whose uncertainty interval is almost symmetric around 0

On August 24, 2018, worldwide media used headlines such as “*No safe level of alcohol consumption, major study concludes*” [1] and “*The ‘safest level of drinking is none’ says alcohol study*” [2] when reporting the findings of a study whose own graph (Fig. 1) showed no statistically significant harmful effect of alcohol on health until consumption increased above one drink per day [3].

The paper only provided relative risks for serious harm, including a 0.5% increased risk at one drink per day, and gave no estimates of the absolute risks from light drinking. However, the press officers at *The Lancet* recognised that such reporting was not in line with the journal’s own guidelines [4] and asked the authors for the absolute figures, which resulted in the following quote: “*914 in 100,000 15–95 year olds would develop a condition in one year if they did not drink, but 918 people in 100,000 who drank one alcoholic drink a day would develop an alcohol-related health problem in a year*” [5].

Putting this into perspective, four extra cases in 100,000 means that, for every 25,000 people having one drink per day, only one more person would experience a (serious) alcohol-related condition each year. Since one standard drink containing 10 g of alcohol per day adds up to 3.65 kg a year, equivalent to 16 bottles (70 cl) of 40% ABV gin, this corresponds to 400,000 bottles of gin shared between 25,000 people to give rise to one case of serious harm. If stood in a line, these bottles would stretch for approximately 40 km, about the length of a marathon.

Viewed this way, the authors’ claim that their results should lead public health bodies “*to consider recommendations for abstention*” [3] looks weakly supported at best. Thus, a risk that was neither statistically nor practically significant became a major headline story – this hardly seems like trustworthy science communication.

**Fig. 2:**
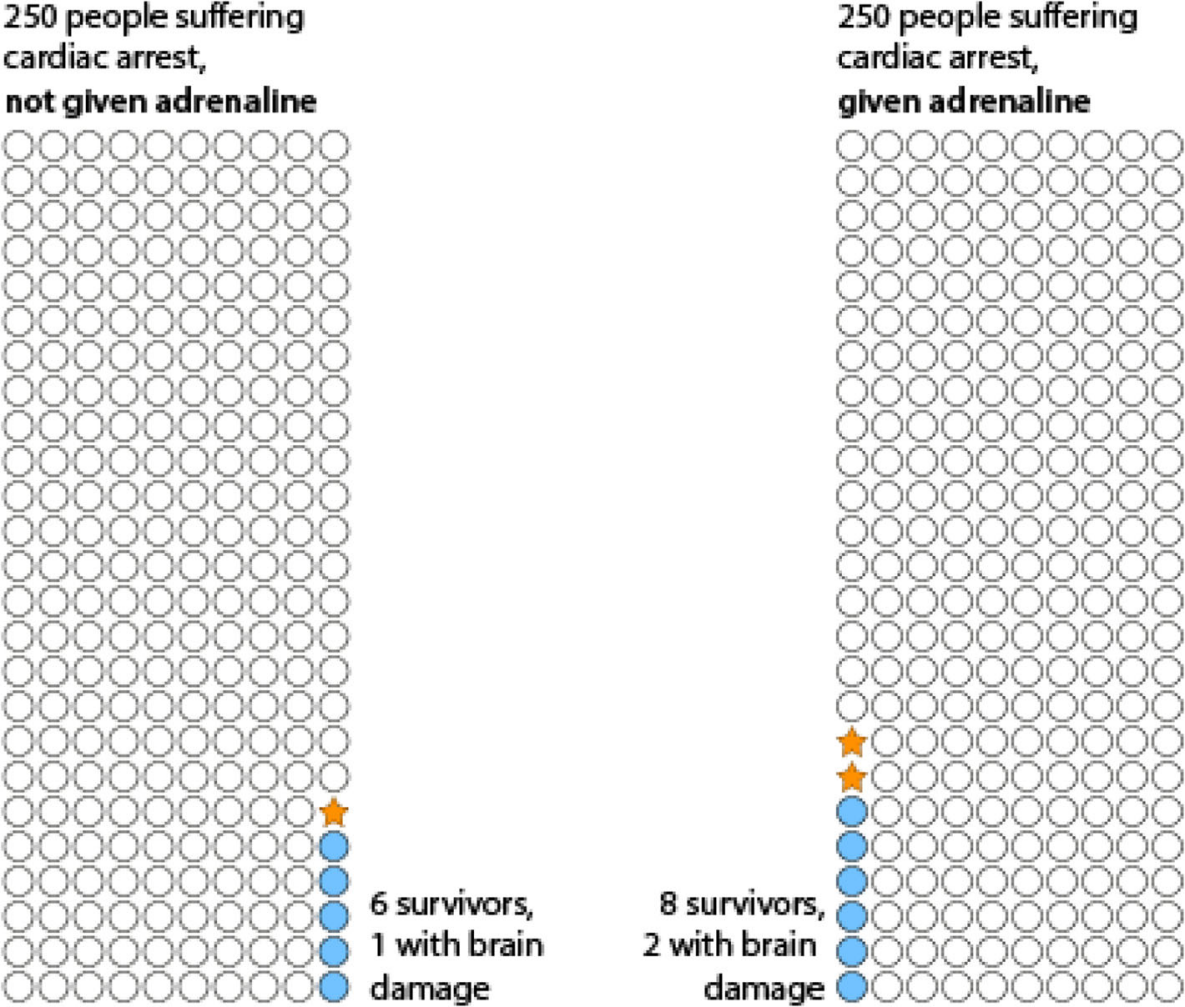
Illustration of the approximate absolute risks in a clinical trial of adrenaline following cardiac arrest

Exaggerated reporting of risk is not restricted to observational data. On July 19, 2018, headlines in the media included “*Cardiac arrest resuscitation drug has needlessly brain-damaged thousands*” [6] and “*Adrenaline ‘doubles risk of brain damage’*” [7] when reporting on an important and large trial that carefully described its results [8]. The paper stated that “*Of 8014 patients included in the primary analysis, 130/4012 (3.2%) assigned to epinephrine compared with 94/3995 (2.4%) assigned to placebo were alive at 30 days*… *More of the survivors in the epinephrine group (39/126 [31.0%]) than in the placebo group (16/90 [17.8%]) had severe neurological impairment (modified Rankin scale score 4 or 5) at hospital discharge*” [8]. Thus, for every 250 people treated with adrenaline, there would likely be two more survivors, one of whom would likely have what the authors defined as “*severe neurological impairment*”. Figure 2 illustrates these findings, indicating that adrenaline appears to be increasing the number of survivors, some of whom may have suffered neurological damage as a result of the cardiac arrest episode. Therefore, these results do not seem to justify the headlines.

## Preventing misinterpretation

In both of the examples provided above, the messages to the public (namely anyone who did not read the actual papers in minute detail) as well as the calls to policy-makers are only weakly related to the actual research results – how did this occur, and how can we prevent it from doing so again (and again)?

The path from research findings to media headlines is often a tortuous one, fraught with various hazards [9]; nevertheless, in the two cases presented above, it is possible to backtrack along the decision-making pathway. Journalists were initially alerted to these two stories by press releases. The alcohol risk study press release included the sub-headline “*The authors suggest there is no safe level of alcohol*” [5], on which the press chose to focus. The adrenaline study press release stated that “*Using adrenaline in cardiac arrests results in less than 1% more people leaving hospital alive – but nearly doubles the survivors’ risk of severe brain damage*” [10], with journalists choosing to literally reproduce the press release. Therefore, should the press officers be held accountable? Did they misinterpret the numbers to ‘spin’ the story? No – the press releases actually quoted the researchers verbatim, with the authors’ own interpretations of the numbers being reported.

These two examples illustrate a seemingly continuing pattern, wherein journalists’ reports are fairly accurately reproduced from the press releases they are given and press officers work hard to clearly and accurately represent their authors’ views. Therefore, much of the responsibility lies with the researchers themselves, perhaps feeling under pressure to maximise the ‘publishability’ of studies.

As the UK Government’s Universal Ethical Code for Scientists states [11], responsible communication is one of the three key responsibilities in scientific research, for good reason. In 1995, research showing that the third generation contraceptive pill doubled the risk of venous thrombosis generated dramatic headlines in the UK and is thought to have resulted in 10,000 abortions (plus 10,000 births) attributed to women stopping the pill [12]. Research on the pill’s thromboembolic effects did indeed show a potential two-fold increase in the risk, yet the absolute risks had changed from only 1 in 7000 to 2 in 7000 [13] – if these numbers had been provided, it is unlikely that such a ‘scare’ would have ensued. Whilst this example may be the most famous, it is not the only case where poor communication of research has resulted in real-life serious consequences.

Media professionals spend every day assessing the consequences of their words, with most health journalists taking that responsibility very seriously. Conversely, researchers working on long and complex projects may not be accustomed to having to consider such potential outcomes. Nevertheless, journals, press offices, the Science Media Centre, the Academy of Medical Sciences and many responsible researchers have produced guidelines to help the accurate communication of risk. Ultimately, it is the responsibility of every researcher and journal editor to consider the wider effects of what they publish, and to publish data true to the results that they have found.

## Conclusions

All those involved in the pipeline of research, publication and publicity have a role in ensuring that risks are clearly presented, putting their magnitude into perspective, without exaggerating their importance, and communicating their uncertainty. We therefore recommend that (a) authors should be able to justify the claims made in their papers and should work closely with press offices in ensuring accurate press releases; (b) journals and peer reviewers enforce guidelines and damp down – rather than encourage – exaggerated claims by authors; (c) press officers ensure that absolute risks are included in press releases and that the conclusions cannot easily be misinterpreted; and (d) journalists demand that researchers put their research claims into perspective.
